# Haemophagocytic lymphohistiocytosis after intravesical BCG administration for bladder cancer presenting with multiorgan failure

**DOI:** 10.1099/acmi.0.000670.v3

**Published:** 2023-09-29

**Authors:** G. D. Liatsos, G. Manousopoulou, A. Poulaki, A. Iliaki, I. Mariolis, D. Vassilopoulos

**Affiliations:** ^1^​ Department of Internal Medicine, "Hippokration" General Hospital, Athens, Greece; ^2^​ 2nd Department of Medicine and Laboratory, School of Medicine, National and Kapodistrian University of Athens, "Hippokration" General Hospital, Athens, Greece

**Keywords:** haemophagocytosis, kidney injury, liver failure, BCG, intravesical

## Abstract

Bacillus Calmette-Guérin (BCG), is administered intravesically as an adjuvant immunotherapy for the treatment of non-muscle invasive bladder cancer. While mild non-infectious problems can occur in up to 85 % of cases, significant local and systemic complications have been reported in 1–5 % of cases. We report the case of a patient with superficial bladder cancer who developed multiorgan failure after intravesical BCG instillation including the kidney and liver with subsequent haemophagocytic lymphohistiocytosis. Our case illustrates the first reported combination of secondary haemophagocytic lymphohistiocytosis with severe renal and liver failure after BCG immunotherapy for bladder carcinoma. Treatment strategy is discussed.

## Data Summary section

No data was generated during this research and none is required for the work to be reproduced.

## Introduction

Bacillus Calmette-Guérin (BCG), is a live attenuated strain of *Mycobacterium bovis* that is administered intravesically as an adjuvant immunotherapy for the treatment of non-muscle invasive bladder cancer [1]. It is usually well tolerated and minor non-infectious side-effects such as fever, malaise, and bladder irritation (dysuria or moderate hematuria) occurring a few hours after intravesical BCG treatment are fairly common (up to 85%) [2], self-limiting (resolving within 48 h) and indicative of a local hypersensitivity reaction [3]. However, significant local and systemic complications can occur in a small percentage of individuals (1–5 %) [2, 3]. Clinical symptoms might present anywhere between hours and years after BCG treatment. The treatment approach is empirical based mainly on the reported experience from case series. Therefore, clinicians may be challenged with more severe systemic complications that occur when BCG organisms gain access to the systemic circulation most likely due to uroepithelial disruption [4].

We report the case of a patient with superficial bladder cancer who developed severe multi-organ failure (MOF) and haemophagocytic lymphohistiocytosis after intravesical BCG instillation.

## Case presentation

A 73-year-old man with a history of hypertension and dyslipidaemia presented with painless, grossly visible hematuria eighteen months before admission. Cystoscopy identified three papillary tumours that were treated with transurethral resection (TUR). Histology revealed a T1 tumour, and imaging investigations ruled out any metastases (Stage 1). Surveillance cystoscopy 6 months later revealed a recurrence of stage one bladder cancer which was followed by second TUR. A month later, weekly BCG induction immunotherapy for 6 weeks was started. However, the third instillation was traumatic, followed by self-limiting dysuria, and the patient refused to complete induction treatment. Five months before admission, he opted to continue with maintenance 3 weekly sessions every 3 months. The first course was unremarkable, but almost a month before admission, and a few hours after the second instillation of the second maintenance course (the eighth instillation in total), he complained of fever, fatigue, and hematuria. His symptoms were initially attributed to bladder infection, and he was treated with amikacin and ciprofloxacin (for three and ten days respectively) without symptom remission.

He was then referred to a district hospital where blood tests revealed acute renal (cr=4.64 mg dl^−1^) and liver failure (ALT=65 U/l, INR=2.0, TBil=21.41 mg dl^−1^, ALP=379 U/l, g-gt=642 U/l) requiring two courses of haemodialysis. Methylprednisolone 80 mg/day and a triple anti-tuberculosis treatment (ATT) with isoniazid, rifampicin, and ethambutol was initiated, only to be discontinued 3 days later due to significant biochemical and clinical deterioration (creatinine=5.36, TBIL=25.66). He was then transferred to our hospital twenty days after his last BCG instillation. On admission, patient was lethargic and jaundiced while his labs showed stage three acute kidney injury (AKI), marked hyperbilirubinemia with moderate cholestasis, and pancytopenia (Table 1). His peripheral blood smear showed marked anisopoikilocytosis, toxic granulation, stacking of erythrocytes with basophilic stippling, and erythroblasts (~8 % of nucleated erythrocytes in different maturation stages). An abdominal ultrasound with Doppler imaging revealed only hepatosplenomegaly while a chest CT showed bibasilar alveolar consolidations with air bronchograms and bilateral effusions.

**Table 1.** Laboratory findings at admission, during hospitalization and follow-up

Blood tests (normal values)		Admission	Day 13	Day 20 ATT Re-initiation	Discharge	2 months after discharge
WBC	>5,200/μl	3.000	3930	4910	3400	3950
Pol/Lypmh	%	71/20	69/17	74/16	62/22	57/26
Hb	>14 g dl^−1^	8.1	8.1	9.9	10.1	10.9
MCV	fL	95.3	98.3	94.1	92.2	97.9
MCH	pg	30.3	32.6	31.1	30.5	33
PLT	>130,000/μl	77 000	174000	140000	115000	163000
Urea	<55 mg dl^−1^	305	166	105	82	41
Creatinine	<1.2 mg dl^−1^	6.1	1.7	1.5	1.2	1.2
LDH	<220 U/l	1109	240	160	279	156
AST	<34 U/l	64	277	40	28	23
ALT	<55 U/l	17	374	164	24	16
ALP	<150 U/l	267	343	232	167	109
ggt	<64 U/l	393	605	366	182	34
TBIL	<1.2 mg dl^−1^	23.40	4.2	2.5	1.80	0.51
DBIL	<0.5 mg dl^−1^	18,20	3.0	1.8	1.0	0.31
Tgs	<150 mg dl^−1^	528	253	n/a	n/a	n/a
Ferritin	<145 ng ml^−1^	14821.39	8348.98	n/a	2292.42	n/a
G6PD	>8 u/grHb	2.7	n/a	n/a	n/a	n/a
Haptoglobulin	>15 mg dl^−1^	<8	n/a	n/a	n/a	n/a
Reticulocytes	<2.5 %	first 3 days low,	then 11–13 %		n/a	n/a
PT time/INR	<14 sec/<1.2	28.7/2.7	14.1/1/3	13.1/1.2	10.9/1.0	n/a
Fibrinogen	200 mg dl^−1^	153	107	308	360	n/a
Blood ammonia	<123 µg dl^−1^	117	n/a	n/a	n/a	n/a

**Fig. 1.**
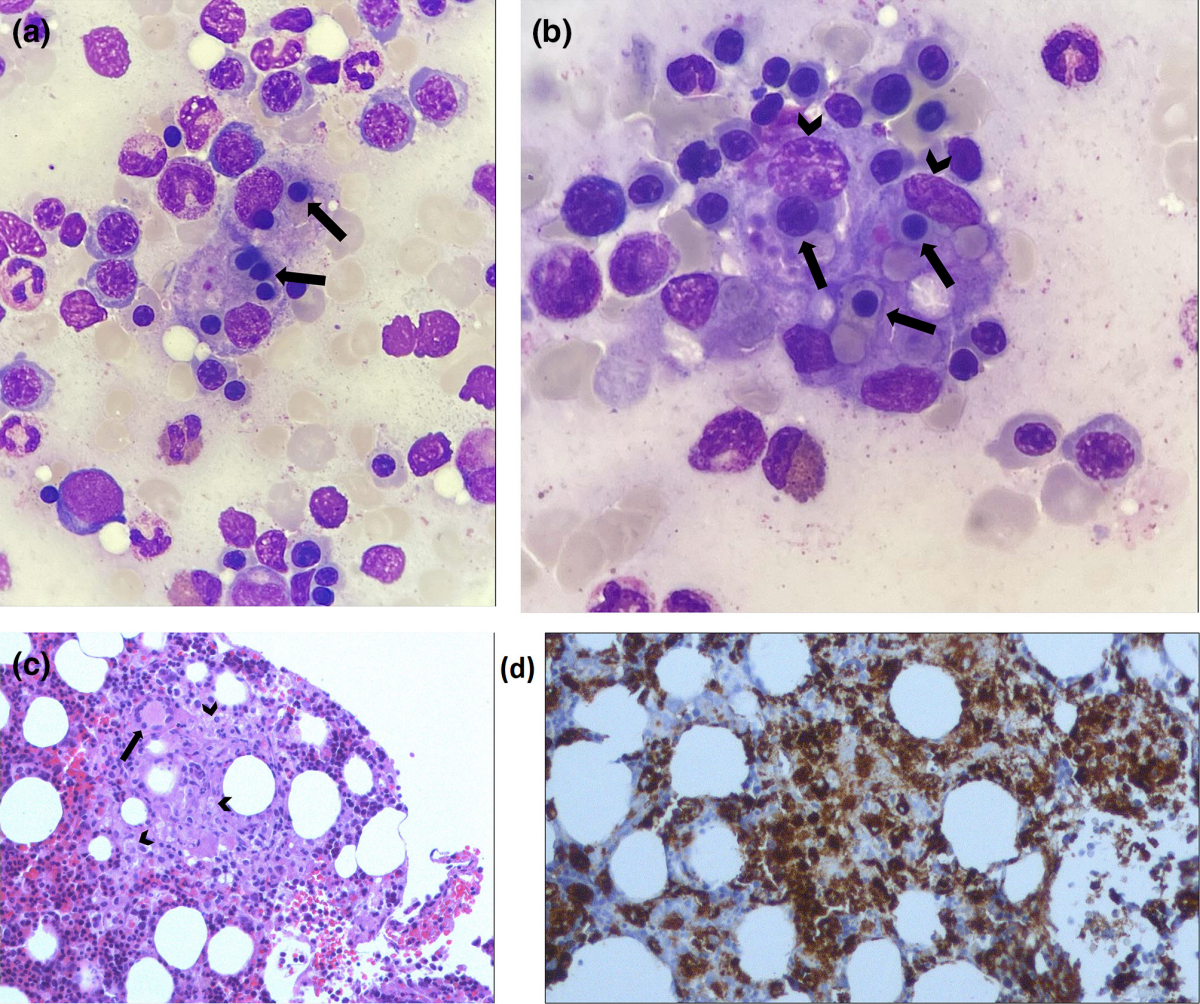
(a). May-Grunwald-Giemsa stain of patient’s bone marrow aspirate showing active haemophagocytosis (arrows). Erythroid hyperplasia, with mild reactive dysplasia, at several maturation stages can be observed. Reversal of the normal myeloid/eryhroid ratio is also shown. Of note, myeloid lineage is maturing without morphologic abnormalities. Light microscopy, ×100. (b). May-Grunwald-Giemsa stain of patient’s bone marrow aspirate showing active haemophagocytosis of erythroblasts (arrows) from bone marrow stromal cells (arrow heads). Marked erythroid hyperplasia and reversal of the normal myeloid to erythroid ratio is again depicted. Light microscopy, ×100. (c). Hematoxylin-eosin stained histologic section of bone marrow biopsy specimen showing a well-formed granuloma with several histiocytes (arrow heads) and a Langerhans giant cell (arrow). Light microscopy, ×40. (d). Histologic section of bone marrow biopsy specimen stained for CD68/KP-1 showing markedly positive (brown) granuloma histiocytes. Light microscopy, ×40.

He was started on broad spectrum antibiotic coverage with meropenem, daptomycin and tetracycline. Further laboratory workup revealed a markedly increased ferritin, hypertriglyceridemia and hypofibrinaemia. A bone marrow (BM) aspiration and biopsy were performed showing active haemophagocytosis of erythroblasts from BM stromal cells (Fig. 1a, b). Bone marrow histology revealed well-formed non-caseating granulomas with several histiocytes and a Langerhans giant cell (Fig. 1c, d). BM Ziehl-Nielsen staining and cultures in Lowenstein-Jensen medium as well as Mycobacteria Growth Indicator Tube (MGIT) for Mycobacteria were all negative.

The above laboratory findings established the diagnosis of secondary haemophagocytic lymphohistiocytosis (HLHscore=337, probability=99.99 %) and on the second hospital day, the patient was started on high dose methylprednisolone (2 mg/kg/day) and intravenous immunoglobulin (IVIG, 0.4 gm/kg/day for 5 days). By day six his clinical status as well as his laboratory values gradually improved while his blood, urine cultures remained negative and antibiotics were discontinued. On day 13, an increase in the aminotransferase levels was noted (Table 1) probably attributed to rapid steroid tapering, and the patient was given three daily pulses of intravenous methylprednisolone (0.5 gm) with gradual improvement. A second BM aspiration was then performed and an XPERT MTB/RIF ultra assay for detection of *Mycobacterium tuberculosis* complex DNA returned positive (without mutations in the rpoB genefor rifampicin resistance). On day 20, his previous triple anti-tuberculous regimen was gradually reinitiated without any adverse events (considering his G6PD deficiency and recent renal and liver failure).

The patient was discharged home on day 33 in good health on rifampicin, isoniazid, ethambutol, pyridoxine, calcium, and Vitamin D3 supplementation with a gradual taper of his methylprednisolone dose. Throughout his follow-up, his anti-TB therapy was well tolerated (Table 1).

## Discussion

Secondary HLH due to BCG intravesical administration is rare, with only a few cases reported in the literature [5–9]. According to those and the present case it affects males between the ages of 49 and 78, and it manifests as constitutional symptoms hours or days after the last BCG instillation. The patient’s age is comparable with the mean age of bladder cancer patients treated with BCG immunotherapy [2], as well as those complicated with BCGitis [3]. Furthermore, HLH caused by intravesical BCG administration might occur between the first and sixth BCG instillation [5–9], although in our case it appeared immediately after the eighth instillation. In the largest review [3], the median number of instillations before systemic BCGitis developed was 6.0 (Q1-Q3 range: 4.0–9.0). The reason for the wide range of instillations preceding HLH or BCGitis incidence is unknown. The multi-organ systemic manifestations following BCG administration could be the result of either a systemic hypersensitivity reaction to *M. bovis* (as evidenced by non-caseating granulomas in the liver [8] and kidneys [10] in the absence of viable mycobacteria in the respective tissues) or a direct result of a disseminated *M. bovis* infection (as evidenced by viable organisms in different organs and/or positive molecular tests). However, a combination of these two mechanisms may be implicated in some cases, resulting in significant macrophage activation and secondary HLH, as was most likely the case with our patient. BM (6/6 of patients), liver (5/6), kidneys (3/6), and lungs (2/6) or their combination are the most often affected organs in HLH caused by BCG instillation. Diagnosis is mostly based upon clinical suspicion taking into account patient’s history of recent BCG administration in association with histological findings of granulomas and/or microbiological detection of *M. bovis* in affected organs.

Treatment consists of high-dose glucocorticoids (methylprednisolone 2–2.5 mg/kg/day or dexamethasone 10 mg/m^2^) combined with IVIG (0.4 g/kg/day for 5 days) and triple anti-TB therapy (rifampicin, isoniazid and ethambutol for 2 months followed by rifampicin and isoniazid for another 7 months since *M. bovis* present *in BCG* is considered resistant to pyrazinamide) [3]. However, it should be noted that early initiation of triple ATT and especially, before the implementation of IVIG and steroids, in cases with liver or renal involvement, may result in early discontinuation or a switch to an alternative ATT (4/6 of the cases) until biochemistry abnormalities resolve. Of notice, even though mortality associated with HLH secondary to tuberculosis is very high 45–50 % [11], all reported patients with BCG-associated HLH recovered.

The possibility that HLH was not caused by BCG and that something else developed in the patient led to a severe clinical scenario of BCG infection with severe dysregulation due to HLH is remote, as BCG was detected in the patient’s BM and extensive laboratory work-up ruled out other potential aetiologies for secondary HLH. Infectious (including HIV, Hepatitis viruses, Parvovirus B19, *CMV*, *EBV*, *Leishmania*, *Brucella *, *Leptospira *, *Rickettsia* spp., and syphilis serology), rheumatologic (ANA, ANCA autoantibodies, ENA screening, serum complement and immunoglobulin levels) or malignant (full body imaging and negative serum cancer markers) diseases were clinically and laboratory ruled out. The patient had no history of past tuberculosis infection and had never received immunosuppressive therapy.

This is the first case of secondary HLH caused by disseminated *M. bovis* infection following intravesical BCG treatment, with severe MOF (stage 3 AKI necessitating haemodyalisis and liver failure indicated by encephalopathy, INR prolongation, and severe hyperbilirubinemia). In a similar reported case [7] organ manifestations were much milder. The mechanisms involved in BCG-associated HLH with MOF, whether infectious or hypersensitivity forms of BCGitis, are various features of the same illness, and BCG-related sequelae appear to be a continuum, with active infection at one end and hypersensitivity reaction at the other. In any case, prompt diagnosis and early initiation of the appropriate immunomodulatory and anti-mycobacterial treatment is crucial for patient's survival.

## References

[1] Sylvester RJ, van der Meijden APM, Witjes JA, Kurth K (2005). Bacillus calmette-guerin versus chemotherapy for the intravesical treatment of patients with carcinoma in situ of the bladder: a meta-analysis of the published results of randomized clinical trials. J Urol.

[2] Larsen ES, Nordholm AC, Lillebaek T, Holden IK, Johansen IS (2019). The epidemiology of bacille Calmette-Guérin infections after bladder instillation from 2002 through 2017: a nationwide retrospective cohort study. BJU Int.

[3] Pérez-Jacoiste Asín MA, Fernández-Ruiz M, López-Medrano F, Lumbreras C, Tejido Á (2014). Bacillus Calmette-Guérin (BCG) infection following intravesical BCG administration as adjunctive therapy for bladder cancer: incidence, risk factors, and outcome in a single-institution series and review of the literature. Medicine.

[4] Gonzalez OY, Musher DM, Brar I, Furgeson S, Boktour MR (2003). Spectrum of bacille Calmette-Guérin (BCG) infection after intravesical BCG immunotherapy. Clin Infect Dis.

[5] Schleinitz N, Bernit E, Harle JR (2002). Severe hemophagocytic syndrome after intravesical BCG instillation. Am J Med.

[6] Thevenot T, Di Martino V, Lagrange A, Petrella T, Faucher J-F (2006). Granulomatous hepatitis and hemophagocytic syndrome after bacillus Calmette-Guerin bladder instillation. Gastroenterol Clin Biol.

[7] Misra S, Gupta A, Symes A, Duncan J (2014). Haemophagocytic syndrome after intravesical bacille Calmette-Guérin instillation. Scand J Urol.

[8] Manganas K, Angelara M, Bountzona I, Karamanakos G, Toskas A (2022). Secondary Haemophagocytic Lymphohistiocytosis Syndrome (HLH) After Intravesical Instillation of Bacillus Calmette-Guérin (BCG): A Case Report and Review of the Literature. Eur J Case Rep Intern Med.

[9] Nascimento L, Linhas A, Duarte R (2016). Disseminated Bacille Calmette-Guérin disease in immunocompetent adult patients. Braz J Infect Dis.

[10] Mohammed A, Arastu Z (2017). Emerging concepts and spectrum of renal injury following Intravesical BCG for non-muscle invasive bladder cancer. BMC Urol.

[11] Elhence A, Aggarwal A, Goel A, Aggarwal M, Das P (2021). Granulomatous tubercular hepatitis presenting as secondary hemophagocytic lymphohistiocytosis: a case report and systematic review of the literature. J Clin Exp Hepatol.

